# Validation of the Diet Quality Questionnaire in Chinese Children and Adolescents and Relationship with Pediatric Overweight and Obesity

**DOI:** 10.3390/nu14173551

**Published:** 2022-08-29

**Authors:** Huan Wang, Anna W. Herforth, Bo Xi, Zhiyong Zou

**Affiliations:** 1 National Health Commission Key Laboratory of Reproductive Health, Institute of Child and Adolescent Health, School of Public Health, Peking University, Beijing 100191, China; 2 Department of Epidemiology, School of Public Health, Cheeloo College of Medicine, Shandong University, Jinan 250012, China; 3 Department of Global Health and Population, Harvard T.H. Chan School of Public Health, Boston, MA 02115, USA; 4 Division of Human Nutrition and Health, Wageningen University and Research, P.O. Box 230, 6700 AE Wageningen, The Netherlands

**Keywords:** Diet Quality Questionnaire, global dietary recommendations, overweight, obesity, children and adolescents

## Abstract

The low-burden Diet Quality Questionnaire (DQQ) has been developed to rapidly assess diet quality globally. Poor diet is often correlated with body size, and certain dietary risk factors can result in overweight and obesity. We aimed to examine the extent to which the DQQ captured food group consumption among children and adolescents in China, and to understand the association of several new indicators of diet quality scores derived from the DQQ with overweight and obesity, using the 2011 wave of the China Health and Nutrition Survey. The DQQ questions are constructed using sentinel foods—that is, food items that are intended to capture a large proportion of the population consuming the food groups. The overall Global Dietary Recommendations (GDR) score, GDR-Healthy score, and GDR-Limit score are novel indicators of diet quality that reflect dietary risk factors for non-communicable diseases derived from the DQQ questions. Multivariable logistic regression analysis was used to examine the associations of the GDR scores with overweight and obesity in the sample. The DQQ questions captured over 95% of children who consumed the food groups. Additionally, we found that the GDR-Limit score was positively associated with general obesity (odds ratio (OR) = 1.43, 95% confidence interval (CI): 1.17–1.74) and abdominal obesity (OR = 1.22, 95% CI: 1.05–1.43), whereas the overall GDR score was negatively related to general obesity (OR = 0.85, 95% CI: 0.74–0.97). The low-burden DQQ could be a valid tool to assess diet quality for the Chinese pediatric population aged 7–18 years. Poor diet quality, as determined by the GDR-Limit score, is associated with the increased risk of obesity in Chinese children and adolescents.

## 1. Introduction

Diets have shifted dramatically and rapidly in China in recent decades with socio-economic development and urbanization [1,2]. Between 1991 and 2009, higher daily fat intake, lower daily protein intake, and an increasing percentage of energy from fat mainly characterized the diets of Chinese children and adolescents aged 7–17 years [3], which subsequently contributed to the prevalence of pediatric overweight and obesity [3]. More recently, a nationwide study of more than one million Chinese school-aged children and adolescents showed that the mean prevalence of overweight and obesity increased markedly from 5.3% in 1995 to 20.5% in 2014 with economic development, highlighting an additional focus on healthy diets and physical activity [4]. Childhood obesity could not only track into adulthood [5] but also correlate with non-communicable diseases (NCDs) and mortality in early adulthood [6], making pediatric obesity a pressing concern in the prevention of obesity and obesity-related adverse outcomes later in life.

Although increasing work has been conducted on diet quality and obesity, diet quality scores do not always correlate with obesity strongly or in expected directions. The Healthy Eating Index (HEI) was reported to be negatively associated with obesity, whereas diversity-based indices were positively associated with obesity in adults [7]. A systematic review and meta-analysis found no significant associations of dietary diversity score with overweight, obesity, or abdominal obesity in either adults or children [8]. Another systemic review provided convincing evidence on the null association between the HEI and body mass index (BMI) in adults and children [9]. These inconsistent findings might be attributed to the considerable heterogeneity of diet assessment or diverse populations with different demographics and socio-economic contexts. For example, diet quality was mainly assessed from a single 24 h recall, multiple 24 h recalls, or a food frequency questionnaire, and many diet quality indicators were country-specific [7,8,9,10], which complicates diet assessment and limits widespread use. Moreover, some diet quality scores, such as diet diversity scores, are designed to measure specific aspects of diet quality, such as nutrient adequacy, rather than total diet quality or risk factors for obesity or NCDs [11]. When selecting and applying indicators, it is important to use them for the purpose intended. Few indicators have been developed that specifically relate to diet-related NCDs.

Measuring diet quality in the pediatric population has additional challenges and is less well studied. Many diet quality indices in the pediatric population have been developed and modified, yet few indices were validated [12,13]. To better monitor healthy diets globally, simple and feasible approaches are highly required to be uniform and standardized, which promotes comparability over time and across countries [14].

Recently, a low-burden Diet Quality Questionnaire (DQQ) entirely based on 29 food groups has been developed to collect dietary data, which takes only five minutes to administer using “yes/no” questions about foods or drinks and is easily understood by respondents [11,15,16]. The novel DQQ is designed to rapidly assess diet quality in populations and has been adapted for more than 100 countries. The DQQ has been developed for the general population but so far has been validated and implemented only for adults (aged 15 years and older). The DQQ questions are constructed using sentinel foods—that is, food items which are intended to capture a large proportion of the population consuming the food groups. The DQQ sentinel food items have not yet been validated for use in populations younger than 15 years of age.

The DQQ data can be used to construct several diet quality indicators at the population level, such as the Minimum Diet Diversity for Women, Food Group Diversity Score, and Global Dietary Recommendations (GDR) scores [15]. The GDR scores are constructed to reflect dietary risk factors for NCDs [15]. Thus, these scores may be plausibly associated with obesity, because dietary risks for NCDs and for obesity are similar. In particular, we would expect that the GDR-Limit score may be associated with obesity, because it captures the consumption of food groups that are generally energy-dense and high in fat and/or sugar.

The present study has two aims, using data from the 2011 wave of the China Health and Nutrition Survey (CHNS). Firstly, we aim to validate the DQQ sentinel foods for Chinese children and adolescents aged 7–18 years. Secondly, we examine the associations of the GDR scores with overweight and obesity. The motivation of these analyses is to understand whether the DQQ could be applied for pediatric use in China, and to understand the relationship of GDR scores (indicators derived from the DQQ) with obesity in children and adolescents.

## 2. Materials and Methods

### 2.1. Study Population

Data were obtained from the 2011 wave of the CHNS, which aimed to understand the interplay of socio-economic transition and nutrition and health-related outcomes in China [17]. The CHNS is an international collaborative project between the Carolina Population Center at the University of North Carolina at Chapel Hill and the National Institute for Nutrition and Health at the Chinese Center for Disease Control and Prevention. This study was approved by corresponding institutional review committees and all participants provided written informed consent for inclusion before they participated in the survey [18]. Further information on the survey design and the publicly available datasets can be found in the cohort profile [17] and at the CHNS website [19].

**Figure 1:**
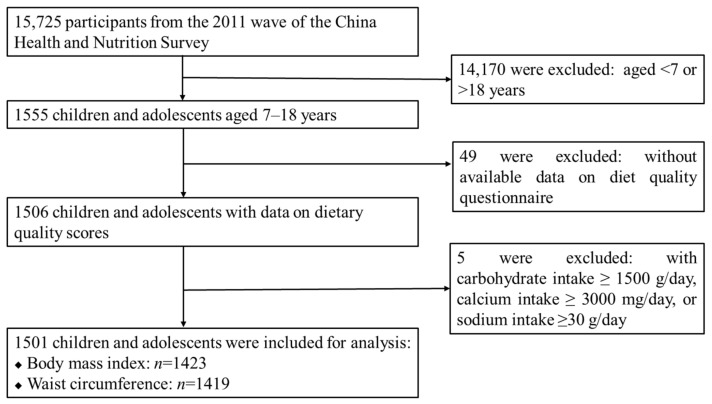
Flow chart of the inclusion/exclusion of participants.

There were 15,725 participants in the 2011 wave of the CHNS, and we included all children and adolescents (hereafter referred to as “children”) aged 7–18 years with complete records on diet and anthropometrics (*n* = 1506). After further exclusion of those with implausible dietary intakes (carbohydrate intake ≥1500 g/day, calcium intake ≥3000 mg/day, or sodium intake ≥30 g/day), a total of 1501 children were included in this cross-sectional study (Figure 1).

### 2.2. Dietary Data Collection

The quantitative dietary data were collected using 24 h dietary recall by trained investigators for three consecutive days that were randomly allocated from Monday to Sunday. For children younger than 12 years, someone who prepared the food for the household was asked to recall the children’s dietary intakes. More details on the dietary interview have been described elsewhere [1]. Nutrient intakes were calculated mainly using the 2009 Chinese food composition database [20], complemented by the 2018/2019 Chinese food composition databases [21,22].

### 2.3. Dietary Assessment

Food intake of the first day from the consecutive three 24 h dietary recalls was coded into 29 food groups following the DQQ tool. The DQQ has been adapted to represent foods in the Chinese context that could reliably capture the food group consumption for the Chinese population, and the identification of sentinel food items for China has been described elsewhere [16]. The China DQQ and further information are available at the Global Diet Quality Project website [15].

The GDR-Healthy score, GDR-Limit score, and overall GDR score were constructed from the dietary intake data: (1) GDR-Healthy score: reflecting five global recommendations on health-protective foods for healthy diets (fruits and vegetables, beans and other legumes, nuts and seeds, whole grains, and dietary fiber); (2) GDR-Limit score: reflecting six global recommendations on dietary components to limit (total fat, saturated fat, dietary sodium, free sugars, processed meat, and unprocessed red meat); (3) overall GDR score: subtracts the GDR-Limit score from the GDR-Healthy score, and reflects all 11 recommendations. The GDR score and its subcomponents were validated against quantitative intakes aligned with each of the recommendations. Specific food groups included for the GDR-Healthy score, GDR-Limit score, and overall GDR score are presented in Table S1. The GDR-Healthy and GDR-Limit scores ranged from 0 to 9 points and the overall GDR score ranged from −9 to 9 points. A lower overall GDR score, lower GDR-Healthy score, and higher GDR-Limit score indicate poorer diet quality [11].

### 2.4. Physical Examination

Height, weight, and waist circumference (WC) were measured by trained field investigators following standardized procedures, as recommended by the World Health Organization (WHO) [23]. Height (accurate to 0.1 cm) and weight (accurate to 0.1 kg) were measured using calibrated weighing and height scales when participants stood straight in light clothes and without shoes. BMI was calculated as weight (kg) divided by height squared (m^2^). WC (accurate to 0.1 cm) was measured at the midpoint between the lowest rib and the iliac crest using non-elastic tapes in the standing position after normal expiration.

### 2.5. Definitions of Overweight and Obesity

Overweight and general obesity were defined using the sex- and age-specific BMI cut-offs for screening among children and adolescents aged 2–18 years, released by the National Health and Family Planning Commission of China in 2018 [24]. Abdominal obesity was defined as WC values ≥ the sex- and age-specific 90th percentiles for Chinese children [25]. Additionally, according to the WHO BMI for age z-scores [26] and the international WC cut-offs [27], overweight, general obesity, and abdominal obesity were re-defined to test the robustness of the results.

### 2.6. Statistical Analysis

The sentinel food analysis was conducted by ranking all foods in each food group in descending order according to the cumulative frequency of food consumption. Additionally, the percentages of the consumption of sentinel food items compared with all food items in the respective 29 food groups were calculated.

Continuous variables were expressed as means ± standard deviations (age, weight, height, BMI, and WC) or medians ± interquartile ranges (dietary intakes and urbanicity index), and categorical variables were shown as numbers (percentages). The independent *t*-test, Wilcoxon rank test, and Chi-square test were used to compare differences in characteristics between boys and girls.

The Cochran–Armitage test was used to examine trends in the prevalence of overweight and obesity across diet quality scores. The multivariable logistic regression analyses were used to evaluate the associations of the GDR scores with overweight and obesity; adjusted odds ratios (OR) and 95% confidence intervals (CI) were estimated after adjusting for sex, age, residence, and urbanicity index. Stratified analyses by sex (boys vs. girls), age (7–12 vs. 13–18 years), and residence (rural vs. urban) were conducted. All analyses were performed using SAS 9.4 (SAS Institute, Cary, NC, USA). Two-sided *p* values < 0.05 were considered statistically significant.

## 3. Results

### 3.1. Participant Characteristics

A total of 1501 children from the 2011 wave of the CHNS with a mean age of 11.7 years were included in this study, 59.9% of whom lived in rural areas. Approximately one tenth of children were overweight (11.0%) or obese (10.0%) and approximately one fifth had abdominal obesity (20.1%). Boys (51.7% of the sample) had significantly higher levels of weight, height, BMI, and WC than girls; boys also consumed more energy, carbohydrates, protein, and fat per day than girls. Boys were more likely to be overweight or obese than girls, whereas there was no significant difference in the prevalence of abdominal obesity between boys and girls (Table 1).

### 3.2. DQQ Sentinel Food Validation for Children and Adolescents

**Figure 2:**
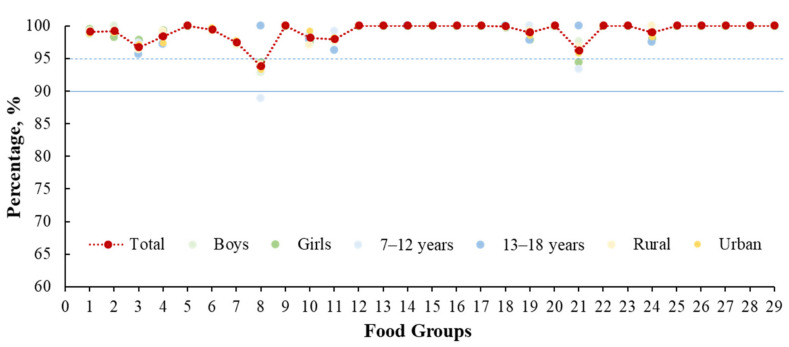
Percentage (%) of the consumption of sentinel food items compared with all food items in respective 29 food groups by sex, age, and residence. Note: 1: Staple foods made from grains; 2: Whole grains; 3: White root/tubers; 4: Legumes; 5: Vitamin A-rich orange vegetables; 6: Dark green leafy vegetables; 7: Other vegetables; 8: Vitamin A-rich fruits; 9: Citrus; 10: Other fruits; 11: Grain-baked sweets; 12: Other sweets; 13: Eggs; 14: Cheese; 15: Yogurt; 16: Processed meats; 17: Unprocessed red meat (ruminant); 18: Unprocessed red meat (nonruminant); 19: Poultry; 20: Fish and seafood; 21: Nuts and seeds; 22: Packaged ultra-processed salty snacks; 23: Instant noodles; 24: Deep fried foods; 25: Fluid milk; 26: Sweetened tea/coffee/milk drinks; 27: Fruit juice; 28: Sugar-sweetened beverages (sodas); 29: Fast food.

In almost every food group, the sentinel food items captured over 95% of children aged 7–18 years who consumed the food groups, suggesting that the DQQ was a valid tool to collect the most common food consumption groups of the Chinese children (Figure 2). For example, people who consumed the sentinel food items (rice, noodles, steamed buns, and bread) accounted for 99.1% of those who consumed grains as a staple food (Table S2). For the vitamin A-rich fruits group, persimmon, mango, papaya, cantaloupe, and hawthorn captured 96.9% of children who consumed this food group; however, the sentinel food items only included the first four foods (capturing 93.8% of children). The specific sentinel food items for each food group are shown in Table S2.

### 3.3. Associations of Diet Quality Scores with Overweight and Obesity

**Figure 3:**
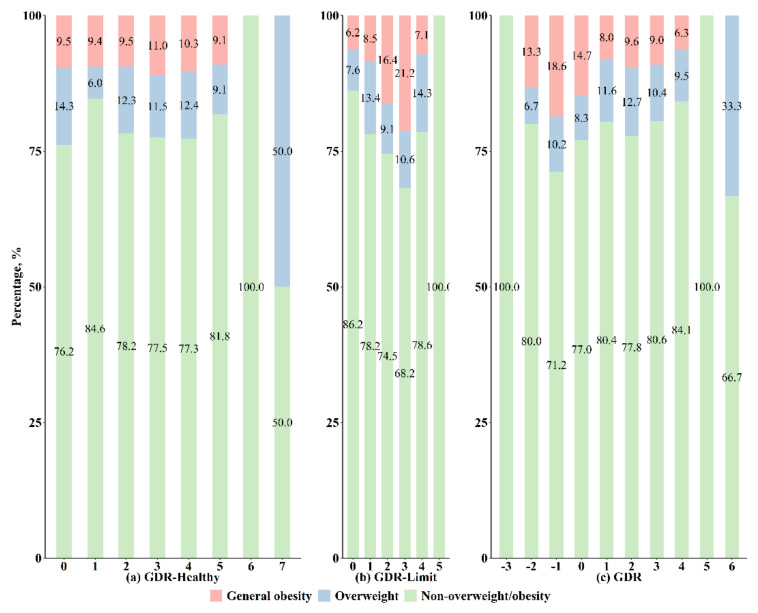
Prevalence of overweight and obesity by Global Dietary Recommendations (GDR) scores.

**Figure 4:**
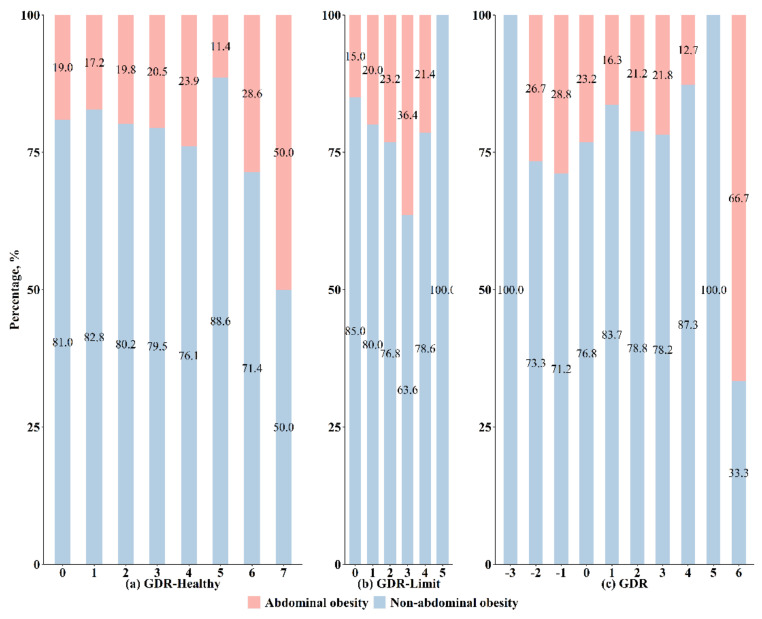
Prevalence of abdominal obesity by Global Dietary Recommendations (GDR) scores.

The prevalence of overweight, general obesity, and abdominal obesity is presented in Figure 3 and Figure 4. Overall, the prevalence of general obesity was higher as the GDR-Limit score increased but was lower as the overall GDR score increased (both *p* for trend < 0.05). The observed trend in the overall GDR score appears to be driven by the GDR-Limit score. The prevalence of abdominal obesity also gradually increased with an increment in the GDR-Limit score (*p* for trend < 0.05). Detailed numbers of overweight and obese children who had each GDR score are shown in Table S3.

After adjustment for sex, age, residence, and urbanicity index, the continuous GDR-Limit score was positively associated with general obesity (OR = 1.43, 95% CI: 1.17–1.74) and abdominal obesity (OR = 1.22, 95% CI: 1.05–1.43), whereas the continuous overall GDR score was negatively associated with general obesity (OR = 0.85, 95% CI: 0.74–0.97) (Table 2). As the diet quality scores were categorized according to their distribution, compared with children with a zero-point GDR-Limit score, those with a GDR-Limit score ≥2-point had increased odds of general obesity (OR = 2.66, 95% CI: 1.53–4.62) and abdominal obesity (OR = 1.60, 95% CI: 1.07–2.40) (Table 2).

**Figure 5:**
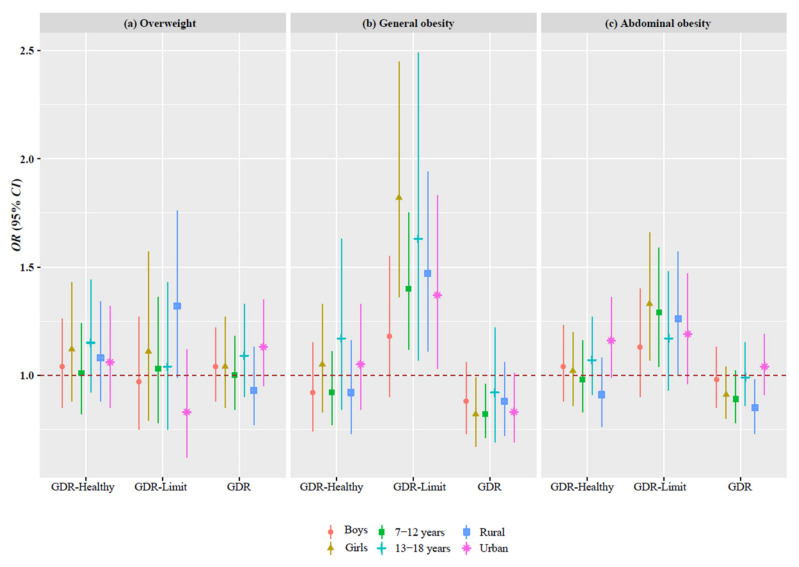
Associations of the Global Dietary Recommendations scores with overweight and obesity by sex, age, and residence. Note: CI, confidence interval; GDR, global dietary recommendations; OR, odds ratio. Logistic regression analyses were used to calculate the odds ratios and 95% confidence intervals with adjustment for sex, age, residence, and urbanicity index.

Stratified analyses by sex (boys vs. girls), age (7–12 vs. 13–18 years), and residence (rural vs. urban) are shown in Figure 5, and similar positive associations between the GDR-Limit score and obesity were found in the subgroups (Figure 5 and Table S4). In the sensitivity analysis, after redefining overweight and obesity, the GDR-Limit score was also positively associated with obesity (Table S5).

## 4. Discussion

In this national cross-sectional analysis of 1501 children and adolescents aged 7–18 years from the 2011 wave of the CHNS, we found that the sentinel foods in the DQQ captured over 95% of children who consumed the food groups, indicating that it is a valid tool for diet quality assessment in this age group. The GDR-Limit score (derived from the DQQ questions) was positively associated with general and abdominal obesity, whereas the overall GDR score was negatively associated with general obesity. The present study suggests that the DQQ tool and new indicators of diet quality are valid for Chinese children and adolescents, and the poor diet quality determined by the GDR-Limit score is associated with the increased risk of obesity.

Our finding that the sentinel food items captured over 95% of the food consumption of children suggests that the China-adapted DQQ tool has the potential to assess the diet quality in the Chinese pediatric population, aligned with global diet quality frameworks in the general population. Although there are several diet quality indicators for Chinese children and adolescents, such as the Chinese Children Dietary Index [28], Chinese Healthy Eating Index [29], and Chinese Healthy Eating Index for School-Age Children [30], these indices rely on 24 h diet recalls and food–nutrient conversion tables, which is time-consuming and poses a heavy burden for both investigators and interviewees. Additionally, these indices only reflect adherence to the Dietary Guidelines for Chinese residents, impeding the comparison of diet quality across countries. Given the increasing attention to adolescent nutrition globally [31,32], aligning with the global framework is useful. Although existing global platforms targeting adolescents are scarce, there are potential global indicators for individuals aged 2–19 years, including the GDR scores calculated easily from the DQQ [14]. Therefore, our study results support the application of the DQQ tool in China and accelerate the process of monitoring healthy diets for children and adolescents at national and global levels.

In our study, we used the GDR scores from the China-adapted DQQ to assess diet quality, which reflects adherence to the WHO Global Dietary Recommendations [11]. We observed higher odds of obesity with a higher GDR-Limit score, and lower odds with a higher overall GDR score. On the other hand, the GDR-Healthy score was not significantly associated with obesity; thus, our findings support the importance of reducing intakes of unhealthy foods as the most important factor for reducing the risk of obesity. The overall GDR score was aligned with the 11 Global Dietary Recommendations on fruits and vegetables, beans and other legumes, nuts and seeds, whole grains, dietary fiber, total fat, saturated fat, dietary sodium, free sugar, processed meat, and unprocessed red meat [11]. Importantly, WHO proposes these recommendations generally based on evidence related to diet-related NCD risks [33,34,35]; therefore, the GDR scores obtained from the DQQ are promising diet quality indicators related to the risk of obesity and other diet-related NCDs in the Chinese population.

This is the first study to validate the DQQ sentinel food items in Chinese children and adolescents. It is also the first study to assess the association between the GDR scores and overweight and obesity in a pediatric population. Several limitations of this study should be noted. First, we only obtained data from the 2011 wave of the CHNS; it is possible that the relationship between diet quality and obesity has changed in the last 10 years. Second, although age, sex, residence, and a comprehensive urbanicity index (proxy of modernization and urbanization) were accounted for in our analyses, residual confounding factors cannot be ruled out, such as physical activity, sedentary behavior, and pubertal development status. Third, although this study finds that the DQQ sentinel foods are valid for children and adolescents, further research is warranted to validate the application of the DQQ in terms of the ability of children and adolescents to reliably report their diet. In general, dietary recall and reporting are challenging for the pediatric age range [36].

## 5. Conclusions

The DQQ sentinel food items could be applied for use in populations aged 7–18 years. The GDR-Limit score is associated with the increased risk of obesity, and the low-burden DQQ could be a valid tool to assess diet quality for Chinese children and adolescents.

## Figures and Tables

**Table 1:** Characteristics of participants by sex.

Characteristics	Total (*n* = 1501)	Boys (*n* = 776)	Girls (*n* = 725)	*p* Value ^b^
Age, years	11.72 ± 3.30	11.75 ± 3.31	11.69 ± 3.28	0.725
Weight, kg	41.56 ± 19.76	43.13 ± 16.38	39.89 ± 22.69	0.002
Height, cm	147.13 ± 17.29	149.24 ± 18.59	144.90 ± 15.50	<0.001
BMI, kg/m^2^	18.42 ± 3.93	18.66 ± 3.88	18.17 ± 3.96	0.020
WC, cm	65.06 ± 14.01	66.21 ± 12.64	63.85 ± 15.23	0.002
Energy, kcal/day ^a^	1525.20 ± 858.45	1657.62 ± 926.57	1412.82 ± 781.48	<0.001
Carbohydrate, g/day ^a^	214.04 ± 138.98	234.58 ± 142.68	193.15 ± 122.90	<0.001
Protein, g/day ^a^	53.73 ± 34.41	59.02 ± 35.15	49.63 ± 31.45	<0.001
Fat, g/day ^a^	49.65 ± 45.55	52.92 ± 46.20	45.32 ± 44.05	<0.001
Urbanicity index ^a^	73.84 ± 35.76	71.29 ± 35.79	76.30 ± 36.06	0.366
Residence, *n* (%)				0.935
Rural	899 (59.89)	464 (59.79)	435 (60.00)	
Urban	602 (40.11)	312 (40.21)	290 (40.00)	
BMI categories, *n* (%)				0.001
Non-overweight/obesity	1125 (79.06)	552 (75.41)	573 (82.92)	
Overweight	156 (10.96)	100 (13.66)	56 (8.10)	
General obesity	142 (9.98)	80 (10.93)	62 (8.97)	
WC categories, *n* (%)				0.651
Non-obesity	1134 (79.92)	586 (80.38)	548 (79.42)	
Abdominal obesity	285 (20.08)	143 (19.62)	142 (20.58)	

BMI, body mass index; WC, waist circumference. Continuous variables are expressed as means ± standard deviations, and categorical variables as numbers (percentages). ^a^ Continuous variables are expressed as medians ± interquartile ranges. ^b^ Differences in characteristics between boys and girls were tested by independent *t*-test, Wilcoxon rank test, or Chi-square test.

**Table 2:** Associations of Global Dietary Recommendations scores with overweight and obesity.

Scores	Overweight		General Obesity		Abdominal Obesity	
	OR (95% CI)	*p* Value	OR (95% CI)	*p* Value	OR (95% CI)	*p* Value
GDR-Healthy						
Continuous	1.07 (0.92–1.24)	0.393	0.98 (0.83–1.15)	0.812	1.03 (0.91–1.16)	0.644
Categories						
≤1	1.00 (Ref.)		1.00 (Ref.)		1.00 (Ref.)	
2	1.86 (1.05–3.29)	0.034	1.01 (0.59–1.73)	0.973	1.13 (0.76–1.69)	0.553
≥3	1.72 (0.99–2.99)	0.056	1.05 (0.63–1.73)	0.863	1.17 (0.80–1.71)	0.419
GDR-Limit						
Continuous	1.03 (0.84–1.27)	0.797	1.43 (1.17–1.74)	<0.001	1.22 (1.05–1.43)	0.012
Categories						
0	1.00 (Ref.)		1.00 (Ref.)		1.00 (Ref.)	
1	1.69 (1.05–2.70)	0.030	1.33 (0.78–2.26)	0.296	1.23 (0.86–1.77)	0.262
≥2	1.21 (0.69–2.11)	0.516	2.66 (1.53–4.62)	0.001	1.60 (1.07–2.40)	0.022
Overall GDR						
Continuous	1.04 (0.91–1.18)	0.572	0.85 (0.74–0.97)	0.016	0.94 (0.86–1.04)	0.244
Categories						
<0	1.00 (Ref.)		1.00 (Ref.)		1.00 (Ref.)	
0	0.87 (0.36–2.14)	0.769	0.89 (0.44–1.80)	0.750	0.81 (0.46–1.44)	0.480
≥1	1.17 (0.59–2.70)	0.554	0.53 (0.29–0.98)	0.043	0.63 (0.39–1.04)	0.070

CI, confidence interval; GDR, global dietary recommendations; OR, odds ratio; Ref, reference group. Logistic regression analyses were used to calculate the odds ratios and 95% confidence intervals with adjustment for sex, age, residence, and urbanicity index.

## Data Availability

The dataset in the present study was open-access and can be freely obtained from the CHNS website: https://www.cpc.unc.edu/projects/china/data/datasets/data_downloads/longitudinal (accessed on 20 November 2021).

## Supplementary Materials

The following are available online at https://www.mdpi.com/article/10.3390/nu14173551/s1, Table S1: Global Dietary Recommendations scores constructed from the Diet Quality Questionnaire; Table S2: Percentage (%) of the consumption of sentinel food items compared with all food items in respective 29 food groups by sex, age, and residence; Table S3: Number and percentage (%) of the overweight and obesity by the Global Dietary Recommendations (GDR) scores; Table S4: Subgroup analysis of associations between the Global Dietary Recommendations scores and overweight and obesity by sex, age, and residence; Table S5: Sensitivity analysis of associations between the Global Dietary Recommendations scores and overweight and obesity.
