# Supplementary material for: Validation of the Diet Quality Questionnaire in Chinese Children and Adolescents and Relationship with Pediatric Overweight and Obesity
Source: Nutrients. 2022 Aug 29;14(17):3551. doi: 10.3390/nu14173551 (PMC9460768; doi:10.3390/nu14173551)
Supplement: Supplementary file 1 [file nutrients-14-03551-s001.zip › nutrients-1733839-supplementary.pdf]

Supplementary Materials

Table S1. Global Dietary Recommendations scores constructed from the Diet Quality Questionnaire.

Food Groups	GDR-Healthy (0 to 9)	GDR-Limit (0 to 9)	Overall GDR [a] (-9 to 9)
1. Staple foods made from grains			
2. Whole grain	√		√
3. White root/tubers			
4. Legumes	√		√
5. Vitamin A-rich orange vegetables	√		√
6. Dark green leafy vegetables	√		√
7. Other vegetables	√		√
8. Vitamin A-rich fruits	√		√
9. Citrus	√		√
10. Other fruits	√		√
11. Grain-baked sweets		√	√
12. Other sweets		√	√
13. Eggs			
14. Cheese			
15. Yogurt			
16. Processed meat [b]		√	√
17. Unprocessed red meat (ruminant)		√	√
18. Unprocessed red meat (non-ruminant)		√	√
19. Poultry			
20. Fish and seafood			
21. Nuts and seeds	√		√
22. Packaged ultra-processed salty snakes		√	√
23. Instant noodles		√	√
24. Deep fried foods		√	√
25. Fluid milk			
26. Sweet tea/coffee/milk drinks			
27. Fruit juice			
28. Sugar-sweetened beverages		√	√
29. Fast food		√	√

GDR, Global Dietary Recommendations. "√" represents the selected food groups used to construct the GDR scores. [a] The overall GDR score is calculated as GDR-Healthy score subtracting GDR-Limit score. [b] Processed meat is double weighted.

Table S2. Percentage (%) of the consumption of sentinel food items compared with all food items in respective 29 food groups by sex, age, and residence [a].

Food groups	Sentinel food items	Total	Boys	Girls	7–12 years	13–18 years	Rural	Urban
Group 1: staple foods made from grains	Rice, noodles, steamed buns, bread	99.1	98.7	99.6	99.1	99.2	99.3	98.8
Group 2: whole grains	Millet, cornmeal, corn, whole wheat bread, oats, barley, black rice	99.2	100.0	98.3	99.3	99.0	99.3	99.0
Group 3: white root/tubers	Potato, lotus root, starch noodles, turnip, yam, taro	96.7	95.6	97.9	97.4	95.6	96.4	97.1
Group 4: legumes	Bean curd or tofu, bean curd sheet, soybean milk, soybeans, other dried beans	98.4	97.4	99.4	99.2	97.3	99.2	97.4
Group 5: vitamin A-rich orange vegetables	Carrots, pumpkin or butternut squash, sweet potatoes that are orange inside	100.0	100.0	100.0	100.0	100.0	100.0	100.0
Group 6: dark green leafy vegetables	Chinese cabbage, water spinach, Chinese spinach, rape, bok choy, sweet potato leaves, broccoli, mustard leaves, chrysanthemum leaves, radish leaves, amaranth leaves	99.4	99.3	99.5	99.4	99.4	99.2	99.7
Group 7: other vegetables	Cabbage, tomatoes, eggplant, loofah, cucumber, local celery, mushrooms, lettuce, radish, bean sprouts, cauliflower, bamboo shoot, bell pepper, seaweed, green beans	97.4	97.4	97.5	97.6	97.2	97.2	97.8
Group 8: vitamin A-rich fruits	Persimmon, cantaloupe, ripe mango, papaya	93.8	92.9	94.4	88.9	100.0	94.1	93.3
Group 9: citrus	Orange, tangerines, pomelo, grapefruit, kumquat	100.0	100.0	100.0	100.0	100.0	100.0	100.0
Group 10: other fruits	Apple, pear, watermelon, banana, grapes, kiwi, dragonfruit, jujube, longan, wampee, pomegranate, lychee, cherry, peaches	98.2	98.6	97.6	98.4	97.9	97.1	99.3
Group 11: grain-baked sweets	Cakes, cookies, sweet pastries, mooncake, rice dumplings, egg tart	98.0	98.1	97.9	99.2	96.3	97.7	98.3
Group 12: other sweets	Candy, chocolates, popsicles, ice cream, jelly pudding	100.0	100.0	100.0	100.0	100.0	100.0	100.0
Group 13: eggs	Eggs	100.0	100.0	100.0	100.0	100.0	100.0	100.0

Food groups	Sentinel food items	Total	Boys	Girls	7–12 years	13–18 years	Rural	Urban
Group 14: cheese	Cheese	100.0	NA	100.0	NA	100.0	NA	100.0
Group 15: yogurt	Yogurt	100.0	100.0	100.0	100.0	100.0	100.0	100.0
Group 16: processed meat	Ham, bacon or larou, sausages, processed beef product, pork jerky, beef jerky, luncheon meat	100.0	100.0	100.0	100.0	100.0	100.0	100.0
Group 17: unprocessed red meat (ruminant)	Beef, lamb sheep or goat, donkey, organs from these animals	100.0	100.0	100.0	100.0	100.0	100.0	100.0
Group 18: unprocessed red meat (non-ruminant)	Pork, pork organs	99.9	100.0	99.8	99.8	100.0	99.8	100.0
Group 19: poultry	Chicken, duck, goose, pigeon, chicken gizzard	99.0	100.0	97.9	100.0	97.9	99.3	98.6
Group 20: fish and seafood	Fish, seafood/fresh water food	100.0	100.0	100.0	100.0	100.0	100.0	100.0
Group 21: nuts and seeds	Peanuts, sunflower seeds, chestnuts, walnuts, sesame paste, almonds, watermelon seeds, pumpkin seeds	96.2	97.7	94.4	93.3	100.0	96.8	95.8
Group 22: packaged ultra-processed salty snacks	Chips, shrimp chips	100.0	100.0	100.0	100.0	100.0	100.0	100.0
Group 23: instant noodles	Instant noodles	100.0	100.0	100.0	100.0	100.0	100.0	100.0
Group 24: deep fried foods	Fried bread stick, fried pancake, fried bean curd, fried glutinous rice ball, chicken nugget	99.0	100.0	98.1	100.0	97.5	100.0	98.3
Group 25: fluid milk	Milk, milk powder	100.0	100.0	100.0	100.0	100.0	100.0	100.0
Group 26: sweet tea/coffee/milk drinks	Flavored milk, yakult, nutri-express, bottled tea beverage	100.0	100.0	100.0	100.0	100.0	100.0	100.0
Group 27: fruit juice	Fruit juice, fruit juice beverage	100.0	100.0	100.0	100.0	100.0	100.0	100.0
Group 28: sugar-sweetened beverages (sodas)	Soft drink, sports drink, energy drink	100.0	100.0	100.0	100.0	100.0	100.0	100.0
Group 29: fast food	Kentucky fried chicken, burger, sandwich/Subway, pizza	100.0	100.0	100.0	100.0	100.0	100.0	100.0

NA, not available. [a] Percentages within 90.0–95.0% are highlighted with a light shade, and percentages below 90.0% are highlighted with a dark shade.

Table S3. Number and percentage (%) of the overweight and obese children by the Global Dietary Recommendations (GDR) scores.

Scores	Body mass index						Waist circumference			
	Non-overweight/obesity		Overweight		General obesity		Non-obesity		Abdominal obesity	
	n	%	*n*	%	*n*	%	*n*	%	*n*	%
GDR-Healthy										
0	16	76.2	3	14.3	2	9.5	17	81.0	4	19.0
1	198	84.6	14	6.0	22	9.4	192	82.8	40	17.2
2	363	78.2	57	12.3	44	9.5	373	80.2	92	19.8
3	324	77.5	48	11.5	46	11.0	329	79.5	85	20.5
4	180	77.3	29	12.4	24	10.3	178	76.1	56	23.9
5	36	81.8	4	9.1	4	9.1	39	88.6	5	11.4
6	7	100.0	0	0.0	0	0.0	5	71.4	2	28.6
7	1	50.0	1	50.0	0	0.0	1	50.0	1	50.0
GDR-Limit										
0	306	86.2	27	7.6	22	6.2	301	85.0	53	15.0
1	555	78.2	95	13.4	60	8.5	565	80.0	141	20.0
2	205	74.5	25	9.1	45	16.4	212	76.8	64	23.2
3	45	68.2	7	10.6	14	21.2	42	63.6	24	36.4
4	11	78.6	2	14.3	1	7.1	11	78.6	3	21.4
5	3	100.0	0	0.0	0	0.0	3	100.0	0	0.0
Overall GDR										
-3	100	100.0	0	0.0	0	0.0	1	100.0	0	0.0
-2	80	80.0	2	6.7	4	13.3	22	73.3	8	26.7
-1	42	71.2	6	10.2	11	18.6	42	71.2	17	28.8
0	157	77.0	17	8.3	30	14.7	156	76.9	47	23.2
1	340	80.4	49	11.6	34	8.0	354	83.7	69	16.3
2	325	77.8	53	12.7	40	9.6	327	78.8	88	21.2
3	170	80.6	22	10.4	19	9.0	165	78.2	46	21.8
4	53	84.1	6	9.5	4	6.4	55	87.3	8	12.7
5	11	100.0	0	0.0	0	0.0	11	100.0	0	0.0
6	2	66.7	1	33.3	0	0.0	1	33.3	2	66.7

Table S4. Subgroup analysis of associations between the Global Dietary Recommendations scores and overweight and obesity by sex, age, and residence.

Scores	Overweight		General obesity		Abdominal obesity	
	**OR (95% CI)**	***p* Value**	**OR (95% CI)**	***p* Value**	**OR (95% CI)**	***p* Value**
Boys						
GDR-Healthy	1.04 (0.85-1.26)	0.731	0.92 (0.74-1.15)	0.476	1.04 (0.88-1.23)	0.655
GDR-Limit	0.97 (0.75-1.27)	0.843	1.18 (0.90-1.55)	0.230	1.13 (0.90-1.40)	0.295
Overall GDR	1.04 (0.88-1.22)	0.674	0.88 (0.73-1.06)	0.172	0.98 (0.85-1.13)	0.771
Girl						
GDR-Healthy	1.12 (0.88-1.43)	0.367	1.05 (0.83-1.33)	0.692	1.02 (0.86-1.20)	0.824
GDR-Limit	1.11 (0.79-1.57)	0.536	1.82 (1.36-2.45)	<0.001	1.33 (1.07-1.66)	0.010
Overall GDR	1.04 (0.85-1.27)	0.702	0.82 (0.67-0.99)	0.037	0.91 (0.80-1.04)	0.177
7–12 years						
GDR-Healthy	1.01 (0.82-1.24)	0.936	0.92 (0.77-1.11)	0.400	0.98 (0.83-1.16)	0.852
GDR-Limit	1.03 (0.78-1.36)	0.838	1.40 (1.12-1.75)	0.004	1.29 (1.04-1.59)	0.019
Overall GDR	1.00 (0.84-1.18)	0.954	0.82 (0.71-0.96)	0.011	0.89 (0.78-1.02)	0.106
13–18 years						
GDR-Healthy	1.15 (0.92-1.44)	0.225	1.17 (0.84-1.63)	0.353	1.07 (0.91-1.27)	0.404
GDR-Limit	1.04 (0.75-1.43)	0.825	1.63 (1.07-2.49)	0.023	1.17 (0.93-1.48)	0.189
Overall GDR	1.09 (0.90-1.33)	0.367	0.92 (0.69-1.22)	0.542	0.99 (0.86-1.15)	0.932
Rural						
GDR-Healthy	1.08 (0.88-1.34)	0.445	0.92 (0.73-1.16)	0.472	0.91 (0.76-1.08)	0.262
GDR-Limit	1.32 (0.99-1.76)	0.061	1.47 (1.11-1.94)	0.007	1.26 (1.00-1.57)	0.047
Overall GDR	0.93 (0.77-1.13)	0.456	0.88 (0.72-1.06)	0.174	0.85 (0.73-0.98)	0.028
Urban						
GDR-Healthy	1.06 (0.85-1.32)	0.628	1.05 (0.84-1.33)	0.655	1.16 (0.99-1.36)	0.076
GDR-Limit	0.83 (0.62-1.12)	0.228	1.37 (1.03-1.83)	0.031	1.19 (0.96-1.47)	0.123
Overall GDR	1.13 (0.95-1.35)	0.166	0.83 (0.69-1.01)	0.057	1.04 (0.91-1.19)	0.591

CI, confidence interval; GDR, global dietary recommendations; OR, odds ratio. Logistic regression analyses are used to calculate the odds ratios and 95% confidence intervals with adjustment for sex, age, residence, and urbanization index, except in specific subgroups.

Table S5. Sensitivity analysis of associations between the Global Dietary Recommendations scores and overweight and obesity.

Scores	Overweight		General obesity		Abdominal obesity	
	OR (95% CI)	*p* Value	OR (95% CI)	*p* Value	OR (95% CI)	*p* Value
GDR-Healthy						
Continuous	1.08 (0.94-1.25)	0.260	0.96 (0.80-1.14)	0.626	0.99 (0.89-1.11)	0.873
Categories						
≤1	1.00 (Ref.)		1.00 (Ref.)		1.00 (Ref.)	
2	1.52 (0.92-2.51)	0.104	1.05 (0.60-1.86)	0.862	1.20 (0.82-1.75)	0.356
≥3	1.51 (0.93-2.45)	0.092	1.00 (0.58-1.72)	0.998	1.12 (0.78-1.62)	0.535
GDR-Limit						
Continuous	1.08 (0.90-1.31)	0.401	1.29 (1.05-1.60)	0.018	1.11 (0.95-1.28)	0.193
Categories						
0	1.00 (Ref.)		1.00 (Ref.)		1.00 (Ref.)	
1	1.48 (0.97-2.25)	0.072	1.25 (0.72-2.16)	0.431	1.14 (0.81-1.60)	0.445
≥2	1.31 (0.80-2.16)	0.279	1.99 (1.11-3.57)	0.021	1.31 (0.89-1.91)	0.172
Overall GDR						
Continuous	1.03 (0.91-1.15)	0.668	0.87 (0.76-1.01)	0.061	0.96 (0.87-1.05)	0.349
Categories						
<0	1.00 (Ref.)		1.00 (Ref.)		1.00 (Ref.)	
0	1.09 (0.50-2.41)	0.825	0.81 (0.38-1.74)	0.588	0.93 (0.53-1.63)	0.794
≥1	1.18 (0.59-2.36)	0.637	0.57 (0.29-1.09)	0.089	0.73 (0.45-1.19)	0.211

CI, confidence interval; GDR, global dietary recommendations; OR, odds ratio; Ref, reference group. Logistic regression analyses are used to calculate the odds ratios and 95% confidence intervals with adjustment for sex, age, residence, and urbanization index. Overweight and general obesity are defined using the World Health Organization body mass index for age z-scores; Abdominal obesity is defined using the international waist circumference percentile cutoffs for central obesity in children and adolescents aged 6–18 years.
